# MicroRNAs: Potential Biomarkers and Targets of Therapy in Allergic Diseases?

**DOI:** 10.1007/s00005-019-00547-4

**Published:** 2019-05-28

**Authors:** Krzysztof Specjalski, Ewa Jassem

**Affiliations:** Department of Allergology, Medical University of Gdańsk, Dębinki 7, 80-210 Gdańsk, Poland

**Keywords:** miRNA, Allergy, Asthma, Allergic rhinitis, Atopic dermatitis

## Abstract

MicroRNAs (miRNAs) are small non-coding RNA molecules that are 18–22 nucleotides long and highly conserved throughout evolution. Currently, they are considered one of the fundamental regulatory mechanisms of genes expression. It has been demonstrated that miRNAs are involved in many biologic processes, such as signal transduction, cell proliferation and differentiation, apoptosis and stress responses. More recently, the role of miRNA has also been revealed in numerous immunological and inflammatory disorders, including allergic inflammation. Specific miRNA profiles were demonstrated in asthma, allergic rhinitis and atopic dermatitis. A core set of miRNAs involved in atopic diseases include upregulated miR-21, miR-223, miR-146a, miR-142-5p, miR-142-3p, miR-146b, miR-155 and downregulated let-7 family, miR-193b and miR-375. Most of the involved miRNAs increase secretion of Th2 cytokines (miR-1248, miR-146b), decrease secretion of Th1 cytokines (miR-513-5p, miR-625-5p) or promote differentiation of T cells towards Th2 (miR-21, miR-19a). In asthma miR-140-3p, miR-708 and miR-142-3p play a role in hyperplasia and hypertrophy of bronchial smooth muscle cells. Some single miRNAs or, more probably, their sets hold the promise for their use as biomarkers of atopic diseases. They are also promising target of future therapies.

## Introduction

MicroRNAs (miRNAs) are small non-coding RNA molecules that are 18–22 nucleotides long and highly conserved throughout evolution (Gebert and MacRae 2019). Their role remained unknown till 1990s when Victor Ambros discovered that lin-4 is a short RNA molecule blocking expression of the genes in the nematode *Caenorhabditis elegans* by RNA–RNA interaction (Lee et al. 1993). Currently, miRNA are considered one of the fundamental regulatory mechanisms of gene expression. Having bound to the region of 3′ untranslated mRNA, miRNAs lead to decrease of gene expression by degradation of mRNA or inhibition of translation. Single miRNA can target numerous genes within one cell type and vice versa—the individual gene may be targeted by multiple miRNAs with only moderate effect of one miRNA on one target (Bartel 2009; Lu and Rothenberg 2013). This makes relations between genes expression and miRNAs really complex. It has been demonstrated that miRNAs are involved in many biologic processes, such as signal transduction, cell proliferation and differentiation, apoptosis and stress responses. Their role in regulating of cell cycle was deeply investigated in oncology. For example, expression of several miRNAs was demonstrated to correlate with the histological type of lung cancer, prognosis and response to therapy (Skrzypski et al. 2014). More recently, the role of miRNA has also been revealed in numerous immunological and inflammatory disorders, including allergic inflammation. Recent studies have demonstrated specific miRNA profiles in some allergic conditions and asthma. Further, Rebane and Akdis (2014) have proposed that miRNAs contribute to the development of allergic diseases by influencing Th1/Th2 polarization, promoting chronic inflammation in epithelium and tissue remodelling as well as activating innate immune cells. In this review we discuss selected articles concerning miRNAs in allergic diseases in order to demonstrate role of miRNAs in the pathomechanism and possible application in monitoring and therapy.

## Role of miRNAs in Allergic Diseases

### Allergic Rhinitis

Allergic rhinitis (AR) is the most common allergic disease worldwide with a prevalence of 1–40%, depending on the region and population studied (Brożek et al. 2017). Although tendency to develop AR is inherited, environmental factors play a substantial role in the initiation of the disease. The key feature of AR is an IgE-mediated inflammation of nasal mucosa, characterized by eosinophilic infiltrations and Th1/Th2 cells imbalance. Th2 cytokines have been found essential in the pathomechanism of AR, particularly interleukin (IL)-4 inducing the subsets of Th2 cells and activating IgE production, IL-5 essential for eosinophils differentiation, maturation and survival, IL-13 playing a key role in mucus hypersecretion, inducing and maintaining IgE production and IgE-mediated responses. IL-1, IL-6 and tumour necrosis factor (TNF)-α secreted mainly by monocytes and macrophages also have a proinflammatory role, whereas transforming growing factor (TGF)-β and IL-10 have regulatory and anti-inflammatory effects (Wang et al. 2014; Eifan et al. 2015). As miRNAs exert well-known regulatory effects on functioning of immune system, their expression in AR has been investigated in recent years.

To analyse the impact of miRNA on cytokines production, Shaoqing et al. (2011) compared expression of 421 miRNAs in nasal mucosa of eight patients with perennial allergic rhinitis and eight healthy controls by means of real-time quantitative reverse transcriptase-PCR (qRT-PCR) on microarrays (Exiqon). In AR group two miRNAs (miR-7 and miR-1194) were upregulated and seven downregulated (miR-224, miR-498, miR-187, miR-874, miR-143, miR-886, miR-767). Interestingly, in the previous studies downregulation of miR-224 was associated with inhibition of TGF-β and miR-498—with the functioning CD4^+^ T cells. Thus, the authors concluded that these miRNAs could play a role in development of AR. Suojalekto et al. (2013) investigated 159 young adults including patients with AR, AR and asthma, non-allergic rhinitis as well as healthy controls (life technologies microarrays). Out of 35 miRNAs analysed in nasal biopsies three, including above-mentioned miR-498 (miR-498, miR-155 and miR-205) were upregulated and one (let-7e) was downregulated in groups with AR. These changes correlated with increased levels of Th2 cytokines. Expression of miRNA in patients with non-allergic rhinitis patients did not differ from that in the healthy controls.

Members of let-7 family were shown to be decreased in both animal models and human studies on asthma and allergic rhinitis. On the contrary, their increased levels inhibit the expression of various inflammatory factors in AR by activating JAK1/STAT3 signalling pathway, repressing IL-13 production and inhibiting of SOCS4 gene transcription (Li et al. 2018).

Chen et al. (2010) investigated a panel of 157 miRNAs in mononuclear leukocytes from human umbilical cord blood with elevated IgE (applied biosystems microarrays). It was found that miR-21 and miR-126 were significantly lower in monocytes from children who developed allergic rhinitis. Downregulation of miR-21 and miR-126 was accompanied by upregulated expression of TGF-βR2 on monocytes. The findings were in line with earlier studies, where a significant increase of TGF-βR2 in epithelium was revealed in patients with AR compared to healthy controls. Upregulation of miR-21 was found to downregulate IL12A and TGF-βR2 (Lu et al. 2009).

Wu et al. (2015) investigated expression of miRNAs in extracellular vesicles (exosomes) in nasal mucus in ten patients with severe AR and ten healthy controls by means of qRT-PCR (applied biosystems microarrays). From 366 tested miRNAs 21 were upregulated and 14 downregulated compared to healthy controls. Further analysis revealed that the processes influenced by differently expressed miRNAs involved B-cell receptor signalling pathway, the natural killer cell-mediated cytotoxicity, T-cell receptor signalling pathway, endocytosis, leucocyte transendothelial migration, etc. As transferring genetic material by exosomes to neighbouring cells modify their function, it seems that exosomal miRNAs form an intercellular signalling system. One of the most significantly upregulated molecules was miR-223 involved in eosinophilic inflammation and associated with lower regulatory T-cell numbers (Lu et al. 2013a). It is well known that Treg cells play an important role in development of tolerance during immunotherapy by producing IL-10. Thus, in the future inhibition of miR-223 could be considered an adjuvant treatment during immunotherapy.

### Asthma

Asthma is a chronic inflammatory disease, characterized by hyperresponsiveness and remodelling of the airways, affecting more than 300 million people worldwide (GINA Report 2018). The pathomechanism of this multifactorial disease is still not fully understood. The most common phenotype is eosinophilic inflammation associated with Th2 response and concomitant atopic diseases. However, some patients show neutrophilic, mixed type or paucigranulocytic pattern. The heterogeneity of asthmatic population is a serious limitation of several studies, because every conclusion should only refer to a single phenotype that was investigated and must not be generalized.

Increased serum levels of several miRNAs, including miR-16, miR-21, miR-125b, miR-126, miR-145, miR-146a, miR-148a, miR-221, miR-223, miR-338, miR-485-3p were demonstrated in general asthmatic populations (Liu et al. 2012; Qin et al. 2012; Panganiban et al. 2016; Lacedonia et al. 2017). Soujalehto et al. (2014) found downregulation of miR-18a, miR-126, let-7e, miR-155, miR-224 and upregulation of miR-498, miR-187, miR-874, miR-143, miR- 886-3p in nasal biopsies in asthmatic patients with no differences between allergic and non-allergic phenotype. MiR-296-5p, miR-16-5p, miR-203 and miR-30d-5p were found to correlate with bronchial hyperresponsiveness while miR-155 with fractional exhaled nitric oxide, nasal nitric oxide and IgE (Davis et al. 2017). In mice model of asthma expression of miR-155, miR-21 and miR-18a correlated with Th2 cytokines levels in bronchoalveolar lavage fluid (Xiao et al. 2016). The significance of diverse expression of miRNAs in asthma is debatable. On one hand, Williams et al. (2009) found no difference in airway biopsies between healthy controls and patients with mild asthma, whereas Jardim et al. (2012) revealed 66 different expressions between asthmatics and controls in bronchial epithelial cells culture (including higher of miR-487b, miR-181, let-7f and lower of miR-203), with no relation to disease severity.

Single nucleotide polymorphisms (SNPs) in miRNAs genes may also play some role in the context of asthma development. Homozygous CC genotype of rs2292832 G/C (miR-149) has the highest frequency in asthmatics with coexisting allergic rhinitis. CC genotype of rs2910164 G/C (miR-146a) and TT genotype in rs2292832 C/T (miR-149) are associated with decreased risk of asthma (Su et al. 2011; Hu et al. 2017).

The role of miRNAs in the pathomechanism of asthma could be analysed in three aspects—regulation of smooth muscle cells, epithelium and immune system.

#### miRNAs in Smooth Muscle Cells

In inflammatory diseases like asthma, smooth muscle cells (SMC) contribute to inflammation and airway wall remodelling by hypertrophy and hyperplasia. They are also capable of secreting cytokines and chemokines and have receptors for these mediators. Biomechanical properties of smooth muscle cells are apparently changed in asthmatic patients, suggesting that there is an asthmatic phenotype of these cells (Deshpande et al. 2015).

Recently, Alexandrova et al. (2016) performed a comprehensive analysis of small non-coding RNA expression in SMC in order to evaluate their permanent deregulation after asthma development. The expression of 905 miRNAs in bronchial SMC was compared between asthmatics and non-asthmatics undergoing lung resection due to lung cancer. Expression of 40 miRNAs was significantly different between the groups, including nine upregulated and 31 downregulated. The authors found that 12 deregulated miRNAs targeted 363 mRNAs expressed in SMC. The effect of targeting miRNAs was associated with significantly altered expression of 38 genes. The set included the gene encoding the peroxisome proliferator-activated receptor γ, which is known to be involved in airway inflammatory and remodelling responses (Oh et al. 2009). Another targets were: ITGB3 (integrin subunit β3—playing a role in the pathogenesis of the disease and sensitization to allergens), BMPR2 (bone morphogenic protein receptor type 2—whose altered expression was shown to inhibit proliferation of vascular smooth muscle), PHB (prohibitin, which is responsible for reducing allergic airway inflammation) and adenosine A2b receptor (ADORA2B)—a powerful bronchoconstrictor acting as an inflammatory mediator and participating in airway wall remodelling (Berk et al. 2005; Weiss et al. 2005; Kariyawasam et al. 2008; Agrawal et al. 2012). Finally, PTEN (phosphatase and tensin homolog—regulating cell cycle, and glucocorticoid action during asthma treatment) was also influenced by miRNAs upregulation (Hosgood et al. 2009).

A few studies showed that miRNAs may influence three important component of SMC physiology (Table 1).

**Table 1:** Influence of selected miRNAs on bronchial smooth muscle cells (SMC) in asthma

Altered miRNA	Target	SMC function
miR-140-3p miR-708 (Deshpande et al. 2005a, b) miR-10 (Hu et al. 2014) miR-142-3p (Bartel et al. 2018)	CD38^a^ p13 kinase WNT pathway	Hyperplasia Hypertrophy Remodelling
miR-25 (Kuhn et al. 2010)	Eotaxin RANTES	Secretion of cytokines and chemokines
miR-133 (Chiba et al. 2009)	IL-13 GTPase RhoA^b^	Contraction

*WNT* Wingless/Integrase 1

^a^CD38 is a cell surface regulating calcium concentration and contractility of SMC. It also plays a role in innate immunity and is involved in T- and B-cell function and immune responses to antigen. In mice model CD38^−/−^ organisms did not develop “asthmatic phenotype” following IL-13 and TNF-α challenge

^b^Exposure of smooth muscles on IL-13 and downregulation of miR-133a leads to upregulation of GTPase RhoA. RhoA is involved in contraction of SMC and is significantly augmented in asthma. Thus, miR-133a is regarded as one of the regulators of airway hyperresponsiveness

Further, in human airway smooth muscle cells exposed to cytokine stimulation mimicking airway conditions in asthma miR-145 was found to be highly expressed. Blocking miR-145 with anti-miR resulted in decreased proliferation and migration of SMC in dose-dependent manner, as well as decreased synthesis of collagen I and myosin heavy chain. The target of miR-145 is Kruppel-like factor 4 (KLF4), a member of KLF transcription factor family. Inhibition of miR-145 increases levels of KLF4 and decreases matrix metalloproteinases (MMP)-2 and MMP-9 (Liu et al. 2015).

Let-7 family has been found to be downregulated in allergic diseases. In asthma this group of molecules plays a substantial role by regulating β2 receptors. Lower expression of let-7f attenuates receptor downregulation (Wang et al. 2011).

#### Functional Role of miRNAs in Epithelium

The bronchial epithelium is considered a physical protection against pathogens. In fact it is also an immunity organ secreting cytokines and regulating inflammatory process in asthma. Asthmatic patients show upregulation of miR-19a and downregulation of miR-181-5p in bronchial epithelium. The former is related to cells proliferation through targeting TGF-βR2 mRNA (Haj-Salem et al. 2015). The latter inversely correlates with sputum and bronchial submucosal eosinophilia. The target of miR-181-5p is SPP1, gene encoding osteopontin, what leads to regulation of IL13, IL-1β and CCL11 expression (Huo et al. 2016).

Solberg et al. (2012) compared expression of miRNAs in bronchial epithelium in 16 steroid-naïve asthmatics, 19 steroid-using asthmatics and 12 healthy controls. Twelve out of 16 steroid—naïve patients had different expression pattern of 217 miRNA compared to healthy controls including members of miR-34/449 family (miR-34b-5p and miR-34c-5p) repressed by IL-13. Moreover, in steroid-naïve subjects with asthma, standard treatment with inhaled glucocorticosteroids had only modest effect on miRNA expression.

#### The Role of miRNAs in Eosinophilic Asthma

Eosinophilic asthma results from Th2 type inflammation (“T2-high asthma”). As a consequence, miRNAs associated with secretion of Th2-related cytokines (IL-4, IL-5, IL-13) or activity of T cells and B cells are dysregulated (Table 2).

**Table 2:** Influence of selected miRNAs on immune system in asthma

Altered miRNA	Target	Function
miR-155 (Zhou et al. 2016a; Zhang et al. 2017)	IL-4 IL-5 IL-13 IL-17a CTLA-4 CD4^+^	Enhanced inflammation and mucus secretion Regulation of T-cell activation Influence on proliferative response
miR-210 (Long et al. 2017)		Inhibition of Treg function
miR-181a		Augmenting sensitivity of T cells to peptide antigens
miR-21, miR-19a (Li et al. 2007; Lu et al. 2009, 2011; Sawant et al. 2013; Long et al. 2017)		Promoting differentiation of T cells towards Th2
miR-221-3p (Zhou et al. 2016b)	PTEN	IL-4 upregulation
miR-1248 miR-146a (Panganiban et al. 2012; Yang et al. 2017)		IL-5 upregulation IL-5 inhibition
let-7 family (Polikepahad et al. 2010; Kumar et al. 2011)		IL-13 downregulation
miR-323-3p, miR-181a, miR-26a (Karner et al. 2017)	SMAD2, SMAD3	TGF-β-dependent signalling pathway modulation
miR-513-5p, miR-22-3p, miR-625-5p (Dong et al. 2016)		Inhibition of Th1 cytokines including IL-12, and interferon-γ

Eosinophils are key cells in T2-high asthma. In ex vivo culture model of murine bone marrow-derived eosinophils miR-21 and miR-223 are upregulated (Lu et al. 2013a, b). MiR-21-deficient eosinophil progenitors have an increased apoptosis rate during maturation. MiR-21-deficient mice have reduced eosinophil levels in blood. In contrast, miR-223-deficient eosinophil progenitors proliferate more intensively.

#### The Role of miRNAs in Neutrophilic Asthma

One of the most significant challenges in asthma is neutrophilic endotype. So far, no biomarkers are available to confirm the diagnosis and monitor the disease. The routine asthma treatment is less efficient and the course of the disease is more severe.

In mice model of neutrophilic, infection-induced asthma Kim et al. (2017) found upregulation of miR-21 in homogenized lungs. The authors demonstrated that miR-21 promotes PI3K-mediated phosphorylation and nuclear translocation of pAKT that suppresses HDAC2 levels and leads to steroid insensitivity.

In human neutrophilic asthma upregulation of miR-199a-5p, miR-223-3p, miR-142-3p and miR-629-3p was found in induced sputum. MiR-629-3p is expressed in bronchial epithelium and miR-223-3p and miR-142-3p—in neutrophils, monocytes and macrophages. Level of miR-199a-5p was significantly associated with impaired pulmonary function (Huang et al. 2018). MiR-629-3p induces upregulation of IL-8 in epithelium, thus providing a mechanistic link between increased miR-629-3p expression and airway neutrophilia in patients with severe asthma (Maes et al. 2016).

### Atopic Dermatitis

Atopic dermatitis (AD) is an inflammatory disease of the skin characterized by pruritus as well as typical appearance and distribution of the rash, which follows a chronic relapsing and remitting course. Atopic dermatitis is one of the most common skin diseases affecting up to 20% of children and 2–8% of the adults (Odhiambo et al. 2009; Wollenberg et al. 2018). Approximately 40–60% of patients have concomitant atopic comorbidities including asthma, allergic rhinitis or food allergies (Flohr et al. 2004). Development of AD results from the interaction of numerous genetical and environmental factors. Genetic studies identified a central role of genes associated with skin barrier impairment. Particularly, filaggrin gene mutations resulting in loss of its function are correlated with early onset, more severe lesions and higher number of comorbidities (Brown 2017). On the other hand, immune dysfunction with Th2-mediated inflammation is a crucial phenomenon which recently has become a target for successful biological therapies. In acute lesions, AD is characterized by profound increases of Th2 cells and Th2-related cytokines (IL-4, IL-5, IL-13, IL-31) as well as IL-22 and Th22 cells (Brunner et al. 2017).

Despite clinical significance of AD, there is less literature data on the role of miRNAs in the mechanism of the disease compared to that for asthma or allergic rhinitis. Most of the studies confirm upregulation of miRNAs known for their involvement in asthma or allergic rhinitis (miR-21, miR-146a, miR-203) (Wu et al. 2015). Other miRNAs, like miR-720, are upregulated solely in AD, possibly due to their role in organ-specific functions, such as keratinocyte cell cycle regulation (Chikh et al. 2011). MiR-483-5p was found to be upregulated in the sera of children with AD, but only in cases with other concomitant atopic diseases (Lv et al. 2014). In skin with AD lesions miR-155 was highly upregulated (Sonkoly et al. 2010). Cytotoxic T lymphocyte-associated protein (CTLA)-4, major inhibitory molecule of T-cell responses, was identified as a target for miR-155. Blockade of CTLA-4 maintains or increases allergic responses with eosinophilia and elevated IgE levels, while increased CTLA-4 inhibits allergic inflammation (Hellings et al. 2002; Jen et al. 2007). Interestingly, early development of AD in children was found to be associated with the high expression of miR-146b, miR-21, miR-22 and miR-375 in their mothers’ breast milk (Simpson et al. 2015). This is in line with clinical data indicating high prevalence of atopic disease in the families of children with AD. Given the fact that breast milks miRNAs are stable and biologically active after ingestion the authors have also hypothesized that miRNAs could affect infants’ immune system and regulate maturation of gastrointestinal tract. In genetically predisposed individuals exposure to “pro-allergic” miRNAs could facilitate development of AD.

## MiRNAs as Biomarkers

Allergic diseases are complex, with numerous genetic and inflammatory pathways involved and many phenotypes. In vast majority of cases IgE-mediated allergy can be confirmed with standard tools (skin tests, sIgE measurement, provocation tests) or some novel methods developed in recent years such as basophil activation test or component-resolved diagnostics (Heinzerling et al. 2013; Borres et al. 2016; Hemmings et al. 2018). However, in many cases diagnosis is based on subjective, observational criteria, for example Hanifin and Rajka criteria for atopic dermatitis requiring long-term observation for confirmation of diagnosis. In asthma the diagnosis in based on clinical picture and presence of expiratory airflow limitation variable over time. However, in small children pulmonary function tests are often difficult to perform and diagnosis remains unconfirmed. Moreover, a quick and simple endotyping of individual asthmatic patients is still unmet need that could make it possible to tailor the treatment properly and increase its efficacy. So far, some of the phenotypes of asthma, e.g., neutrophilic, do not have well established biomarkers. Thus, there are objectives not sufficiently achievable with the tools currently applied in clinical practice. Biomarkers facilitating diagnosing of these diseases, their endotyping and/or predicting clinical outcomes and drug response would be a big step forward to reach these goals. The most significant disadvantage of applying miRNAs is their specificity which is uncertain due to potential role in pathomechanism of several diseases.

The ideal biomarker should have good performance characteristics, such as sensitivity, specificity and positive and negative predictive values. Moreover, it should be relatively safe, easy to measure and cost-effective (Parikh and Vasan 2007). Thus, apart from scientific evidence confirming that its use influences disease diagnosing or outcome, the biomarker should be easily applied in the clinical practice. In some aspects miRNAs seem to be a promising direction in this field.

As mentioned above, allergic rhinitis is characterized by typical profiles of miRNAs expression. Among numerous candidates for biomarkers of AR, miR-181a seems to be particularly promising. The serum level of this miRNA is significantly decreased in patients with AR, and its expression negatively correlates with disease severity, levels of osteopontin, Th2 cytokines (IL-4, IL-5) and positively correlates with Th1 cytokines (IL-12, interferon γ) (Liu et al. 2016b). However, due to complex relations between miRNAs and inflammatory pathways in AR, expression of single miRNA may not be a truly reliable biomarker. Thus, several attempts were made to find a model based on a group of selected miRNAs that correlate with the presence and severity of AR. For example, a combination of miR-126-5p, miR-19a-5p and miR-26a-5p could confirm diagnosis of AR with sensitivity of 90% and specificity of 70% and correlates with disease severity (Jia et al. 2018). In other study, seven candidate miRNAs were selected based on earlier studies and investigated in 85 patients with AR and 57 non-atopic patients after adenoid surgery (He et al. 2017). Expression of miR-221 and miR-142-3p was found to be elevated and combination of their levels could confirm diagnosis of AR with the sensitivity of 81% and specificity of 65%. Expression of miR-221 also correlated positively with clinical data (total nasal score, itching and sneezing scores). To conclude, currently diagnosis of allergic rhinitis is based on patients’ history, skin tests and/or IgE determination. miRNAs determination will surely not replace this but the future studies can prove their value in assessment of severity and prognosing.

Asthma is a heterogeneous disease with phenotypes that may react differently to therapy, so there is an apparent need for novel tools to differentiate patients into several subgroups. Milger et al. (2017) pre-identified miRNA biomarkers in murine models of asthma, and candidate biomarkers were confirmed in a test cohort of patients with asthma and healthy controls. Although the observed differences in the single miRNAs were considered small, the combination of five miRNA ratios (miR-21-5p/miR-15a-5p; miR-27a-3p/miR-15a-5p; miR-29c-3p/miR15a-5p; miR-223-3p/miR-425-5p; miR-15a-5p/miR342-3p) was found a reliable asthma biomarker. However, with the use of these biomarkers the authors were not able to determine particular asthmatic phenotypes. Although patients taking oral steroids or receiving antileukotriene therapy had different expression, inflammatory phenotype was not reflected. Panganiban et al. (2016) compared patients with asthma, AR and healthy controls. They found 30 miRNAs with expression significantly different between the groups. Specific for asthma was upregulation of miR-16, miR-223, miR-148a and miR-146a, and downregulation od miR-299-5p, miR-570 and miR-150. The model with six most relevant miRNAs (miR-125b, miR-16, miR-299-5p, miR-126, mir-206, miR-133b) allowed differentiation of healthy subjects, AR or asthma with the sensitivity of 92.4%. It was also possible to find two expression profiles corresponding with peripheral eosinophil levels.

One of the most important challenges would be to find biomarkers predicting treatment outcomes. McGeachie et al. (2017) investigated serum expression of 738 miRNAs in 160 children with asthma aged 5–12 years in search for predictors of asthma remission at the age of 14. The developed model based on 12 variables (including miR-146b-5p, miR-106a, miR-126, miR-30a) allowed prediction of remission with sensitivity of 84% and specificity of 70%. Thus, miRNAs are potentially predictive biomarkers for treatment outcomes.

Finally, Trinh et al. (2017) investigated SNPs in miR-196a (rs11614913 T/C), miR-146a (rs2910164 C/G) and miR-499 (rs3746444 A/G) in 347 Korean asthma patients and 172 healthy controls. The CT/CC genotype of miR-196a rs11614913 was associated with eosinophilic asthma (*p *= 0.004) and a higher sputum eosinophil count compared with the TT genotype (*p *= 0.003). The CG/GG genotype of miR-146a rs2910164 tended to be associated with higher bronchial hyperresponsiveness to methacholine (PC_20_) compared with the CC genotype. The AG/GG genotype of miR-499 rs3746444 was associated with higher predicted values of forced expiratory volume in 1 s (%FEV_1_) compared with the AA genotype (*p *= 0.008).

The presented studies concentrated mostly on finding miRNAs typical for asthma. As this is a heterogenous disease, it would be beneficial to find miRNAs associated with its phenotypes. So far, this is an unmet need. Differentiating between asthma phenotypes on the basis of miRNAs would have practical application in the future.

Another clinically significant application of miRNAs may be associated with their potential predictive value in allergen immunotherapy. Allergen immunotherapy is a method of tolerance induction by administration of increasing doses of allergen to which the patient has IgE-mediated allergy. Currently in clinical practice response to allergen immunotherapy is based mainly on observation of clinical symptoms. For scientific purposes challenge tests are also applied. Although reliable, they are time-consuming and potentially dangerous for the patients, because they involve exposure to clinically relevant allergens. Determination of miRNAs profiles during immunotherapy would provide an insight into its genetic consequences. Thus, some studies have been conducted to assess whether miRNAs monitoring could be an objective method of evaluating the response and prognosing.

In the study of Specjalski et al. (2016) expression of 740 miRNAs was assessed before and 24 h after initial phase of allergen immunotherapy with wasp venom. Expression of five miRNAs changed significantly (miR-370, miR-539, miR-502-3p, miR-299 and miR-29c), and another 62 changed twofold in some patients, including increases in miR-143, let-7d and decreases in proinflammatory miR-301, miR-146b, miR-106, miR-485. In the study of Luo et al. (2016) 24 children with AR and house dust mites allergy were treated either with sublingual (SLIT) or subcutaneous immunotherapy (SCIT). In both groups after 3 months of treatment increased expression of miR-146a correlated positively with Foxp3 mRNA level and negatively with disease severity. Increased IL-10 and decreased of IL-5 levels were also found with no differences between SLIT and SCIT. In none of these studies changing expression of miRNAs was confronted with clinical response to immunotherapy. Such assessment was made by Hou et al. (2015). The authors investigated miRNAs profile in patients with allergy to Japanese cedar pollen undergoing SLIT. Out of 15 patients, six underwent sublingual immunotherapy with the pollens and nine received placebo. During pollinating season seven subjects in the placebo group and none administered SLIT developed symptoms of rhinitis, respectively. Sera miRNAs levels were determined in pre- and post-seasonal samples. The only miRNAs that changed significantly in both, SLIT and placebo group, was miR-223. In placebo group development of pollinosis was significantly associated with downregulation of let-7b. Although monitoring of immunotherapy with reliable biomarker would be useful, currently it seems that no miRNAs could be applied in this setting.

## MiRNAs as Therapeutic Target

MiRNAs have been assessed as therapeutic agents in several in vitro and animal studies. Although their results seem promising, effects of therapy with miRNAs or their antagomirs are difficult to predict. Relations between gene expression and miRNAs are complex. As a result, administration or silencing of a single miRNA could modify expression of numerous genes with unknown consequences. In multifactorial diseases, successful silencing of a single gene may not be efficient in clinical practice. On the other hand, miRNA are still promising therapeutic agents due to the site-specificity which is usually associated with the reduction of side effects. However, the potential of miRNAs-based therapeutics has not been fully applied due to inefficient transportation of miRNA to the target cells. This requires carriers (e.g., viral or lipid-based vectors) which are now extensively investigated.

In allergic diseases, the crucial processes influenced by miRNAs include eosinophils development, T cells differentiation and activation and development and activation of mast cells. Thus, they may be considered as a potential means of modulating gene expression to reduce Th2 inflammation.

In in vitro studies miR-106b was found to negatively regulate pro-allergic properties of dendritic cells, whereas and miR-143, which is downregulated in AR, suppressed IL-13 receptor α1 chain gene expression in nasal epithelial cells (Yu et al. 2013; Tang et al. 2015; Teng et al. 2015). MiR30a-5p targeted suppressor of cytokine signalling 3, playing a role in T helper cell differentiation and involved in the development of AR (Zhao et al. 2018). Administration of miR-375 agomir, by inhibiting JAK2/STAT3 pathway, led to downregulation of IL-6 and TNF-α and upregulation of IL-10 in nasal mucosa cells (Wang et al. 2018).

In animal models, effects of miRNAs administration were investigated mostly in the context of AR. Deng et al. (2015) investigated the influence of miR-135a, which had been known to downregulate GATA-3, playing an important role in mast cells degranulation. MiR-135a was administered intranasally to ovalbumin (OVA)-sensitized mice with the use of lentiviral vector. Following the treatment, infiltrations with eosinophils and mast cells were suppressed in nasal mucosa and Th1/Th2 cells balance in the spleen shifted towards Th1 dominance. Similar effects were observed in the study of Luo et al. (2014). Intranasal application of miR-135a in OVA-sensitized mice lowered expression of both GATA-3 and IL-4. Xiao et al. (2017) found that expression of miR-133b was significantly decreased in nasal mucosa of mice with AR. Its restoration by nasal administration of miR-133b agomir led to amelioration of rhinitis symptoms and decreasing of serum levels of IgE, IL-4, IL-5 and TNF-α. Additionally, miR-133b appeared to attenuate eosinophils and mast cells infiltration in nasal mucosa.

In murine model of asthma Niu et al. (2017) tested effects of miR-33b known for its regulatory role in mediating mast cell function. The study demonstrated that several key elements of asthma, including infiltration of inflammatory cells, especially eosinophils, as well as Th2 cytokines in bronchoalveolar lavage fluid, airway hyperresponsiveness and specific IgE levels were reduced with miR-33b. Thus, miR-33b-mediated inhibition of asthma development was associated with Th2 response inhibition.

Liu et al. (2016a) investigated the role of miR-146a in an enforcing immunotherapy in a mice model of AR. MiR-146a was shown to be downregulated in both humans and mice sensitized to OVA. Sensitized mice with AR symptoms were administered intranasal immunotherapy with nanovaccine (i.e., vaccine containing nanoparticles as carriers or adjuvants) containing either OVA, miR-146a or both (Hayat and Darroudi 2019). The study showed that nanovaccine with ovalbumin exacerbated AR symptoms, whereas there was no impact of miR-146a. However, adding miR-146a to ovalbumin significantly enforced the effect of immunotherapy, leading to inhibition of AR symptoms and increase of Treg cells in nasal mucosa. The effect was at least partly associated with the induction of TGF-β, which is of crucial importance in immunotherapy and induction of IL-10 suppressing allergic reaction in nasal mucosa (Liu et al. 2016a; Luo et al. 2015).

Another possible therapeutic option are antagomirs. They are a class of chemically engineered antisense oligonucleotides complementary to the specific miRNA target. They bind to both miRNAs and miRNAs binding site of mRNA targets. As a consequence, they sequester miRNA in competition with cellular target mRNA leading to functional inhibition of the miRNA and derepression of the direct targets. Depending on antagomir chemistry miRNA is either sequestered in heteroduplex or its degradation is promoted (Stenvang et al. 2012). So far their application has been investigated in animal models. In mice, anti-miR-221 reduced eosinophils in bronchoalveolar lavage (Qin et al. 2012). Anti-miR-145 and anti-miR-126 reduced eosinophilic infiltrations in bronchi, Th2 cytokines production and airway hyperreactivity (Collison et al. 2011; McClure et al. 2014). By contrast, such results were not achieved with anti-miR-155-5p. Although miR-155-5p was highly upregulated in murine asthma, intranasal administration of antagomir had no effect on airway hyperresponsiveness or Th2 cytokines expression (Plank et al. 2015).

In humans some phase I clinical trials with miRNAs and antagomirs have been conducted in recent years. Miravirsen (anti-miR-122) tested in hepatitis C was found to be efficient in decreasing HCV load (Janssen et al. 2013). MiR-16 was tested in mesothelioma and miR-34 in multiple solid tumours. Both were found safe and will be assessed in next phase trials (Hong et al. 2015; Reid et al. 2016).

In relation to allergic diseases the unanswered questions concerning therapy with miRNAs include defining groups of potential good responders, optimal route of administration and safety assessments.

## Conclusions

miRNAs have an unquestionable influence on regulation of allergic inflammation. A core set of miRNAs involved in atopic diseases include upregulated miR-21, miR-223, miR-146a, miR-142-5p, miR-142-3p, miR-146b, miR-155 and downregulated let-7 family, miR-193b and miR-375. Some single miRNAs or, more probably, their sets hold the promise for their use as biomarkers of atopic diseases. They are also promising target of future therapies.

Although our knowledge about role of miRNAs in allergic inflammation has expanded in recent years, several areas warrant future investigation. Notably, except for a few early studies in human asthma or rhinitis, most of the knowledge on the role of miRNAs in allergic inflammation is based on cell cultures and murine models. Thus, future studies should be more focused on human settings. Application of miRNAs as non-invasive biomarkers should be investigated with an emphasis of possible determination of disease endotype and predicting treatment effects. Another aspect is the possible therapeutic application. Considering complex relations between miRNAs and genes with Th1/Th2 balance regulated by numerous miRNAs, results of animal studies cannot be easily translated to the clinics. Combinatorial targeting of a key RNA by several miRNAs would be a promising area of future investigation.
